# Evaluation of semen parameters and histopathological comparison of inguinal and abdominal orchiectomy specimens in late post pubertal men with unilateral undescended testis

**DOI:** 10.1186/s12610-025-00270-5

**Published:** 2025-06-17

**Authors:** Serkan Yenigurbuz, Caner Ediz, Hasan Huseyin Tavukcu, Serkan Akan, Omer Yilmaz

**Affiliations:** 1 Department of Urology, Sultan Abdulhamid Han Education and Research Hospital, Tıbbiye Street, Istanbul, Uskudar 34668 Turkey; 2 Department of Urology, Medipol Hospital, Istanbul, Turkey; 3 Department of Urology, Fatih Sultan Mehmet Education and Research Hospital, Istanbul, Turkey

**Keywords:** Undescended Testis, Orchiectomy, Fertility, Histology, Testicular cancer, Testicule non descendu, Orchidectomie, Fertilité, Histologie, Cancer du Testicule

## Abstract

**Background:**

The embryological development of the testicles and their descent to the scrotum is multifactorial process, and the role of the internal inguinal ring has not yet been clarified. In this study, we aimed to assess the role of the internal inguinal ring in this process by comparing the inguinal and abdominal orchiectomy specimens histopathologically. The patients were classified according to localization of the undescended testicle as group 1 (*n* = 11), consisting of abdominal-localized individuals, and group 2 (*n* = 40), composed of inguinal-localized individuals. Data regarding age, side of the undescended testicle, testicular volume, spermatic cord length, testosterone level, semen analysis, and histopathological reports of undescended testicles in both groups were analyzed and compared.

**Results:**

The median age of groups 1 and 2 was twenty-one. The median testicular volume was significantly lower in group 1 than in group 2 (*p* = 0.002). In addition, progressive motility and normal sperm morphology rates were substantially higher in group 2 than in group 1 (*p* = 0.019 vs *p* = 0.029). There was no difference between the groups in terms of analyses of histopathological parameters performed in testis tissue. (*p* > 0.05).

**Conclusions:**

Although testicular volume, sperm motility, and normal morphology rates were lower in abdominal testes, no significant differences were found in histopathological parameters between the groups.

## Résumé

**Contexte:**

Le développement embryologique des testicules et leur descente vers le scrotum est un processus multifactoriel, et le rôle de l’anneau inguinal interne n’a pas encore été clarifié. Dans cette étude, nous avons cherché à évaluer le rôle de l’anneau inguinal interne dans ce processus, en comparant histopathologiquement les échantillons d’orchidectomie inguinale et abdominale. Les patients ont été classés en fonction de la localisation du testicule non descendu en groupe 1 (*n* = 11), composé d’individus avec localisations abdominales, et en groupe 2 (*n* = 40), composé d’individus avec localisations inguinales. Les données concernant l’âge, le côté du testicule non descendu, le volume testiculaire, la longueur du cordon spermatique, le taux de testostérone, l’analyse du sperme et les rapports histopathologiques des testicules non descendus dans les deux groupes ont été analysées et comparées.

**Résultats:**

L’âge médian des groupes 1 et 2 était de vingt et un ans. Le volume testiculaire médian était significativement plus faible dans le groupe 1 que dans le groupe 2 (*p* = 0,002). La mobilité progressive et les pourcentages de spermatozoïdes avvec une morphologie normale étaient nettement plus élevés dans le groupe 2 que dans le groupe 1 (*p* = 0,019 vs *p* = 0,029). Aucune différence strtistique n’a été observée entre les groupes en termes d’analyses des paramètres histopathologiques effectuées dans le tissu testiculaire.

**Conclusions:**

Bien que le volume testiculaire, la motilité des spermatozoïdes et les taux de morphologie normale étaient plus faibles dans les testicules abdominaux, aucune différence significative n’a été observée dans les paramètres histopathologiques entre les groupes.

## Introduction

An undescended testicle, one of the most common abnormalities in newborns, is a condition in which one or both testicles are not in the usual scrotal position and are located elsewhere between the abdomen and upper scrotum. It can be seen in up to 9% of standard term births and 30% of preterm births uni-or bilaterally [1]. Nearly 80% of the undescended testicles descend to the scrotum within the third month of life. Thus, real incidence drops to approximately 1% [2].

Gonadotropin deficiency or unresponsiveness, testosterone deficiency, defective testosterone synthesis, defects in testicular development, anatomical reasons, chromosomal abnormalities, congenital syndromes, and iatrogenic injuries can be responsible for an undescended testicle [3–5].

Testicular descent is a two-stage process. The initial transabdominal stage, from 10-15 weeks' gestation, involves the regression of the cranial gonadal suspensory ligament, moving the testes to the internal inguinal ring by 12 weeks. A pause occurs here (15–28 weeks) as the processus vaginalis and gubernaculum create a path through the inguinal canal to the scrotum. The second, rapid inguinoscrotal stage then occurs between 28 and 35 weeks' gestation [6, 7]. Concurrently, testosterone shortens the elongated and masculinized gubernaculum by reducing its viscoelasticity. During the passage through the inguinal canal, the testis may be located in the abdominal cavity or at any level of the inguinal canal as an undescended testis. If this is not corrected surgically in the early period, atrophy in the seminiferous tubules precludes spermatogenesis and histogenesis of spermatogenesis.

To our knowledge, one study assessing the unknown points of the testicular descent in the inguinal canal by examining orchiectomy specimens of adults exists in English literature [8]. Therefore, comparing the testis specimens before and after the inguinal canal can contribute to the literature regarding the effects of the inguinal canal on this process. This study aims to assess the role of the inguinal canal by comparing the abdominal and inguinal testis specimens and semen parameters in adult patients of similar age groups who underwent an orchiectomy procedure with the diagnosis of an undescended testicle.

## Patients and methods

The local ethics committee approved This descriptive retrospective study conducted by the World Medical Association Declaration of Helsinki's "Ethical principles for medical research involving human subjects" (Ethical committee approval number: HKAEK/20-58).

### Patient population

Data from the patients who underwent an orchiectomy procedure in our clinic with the diagnosis of undescended testicles between January 2010 and December 2018 were retrospectively examined. Fifty-one patients with available data were enrolled in the study out of 61 recordings. The patients were classified according to localization of the undescended testicle as group 1 (*n* = 11), consisting of abdominal-localized individuals, and group 2 (*n* = 40), composed of inguinal-localized individuals.

### Inclusion and exclusion criteria

**Fig. 1:**
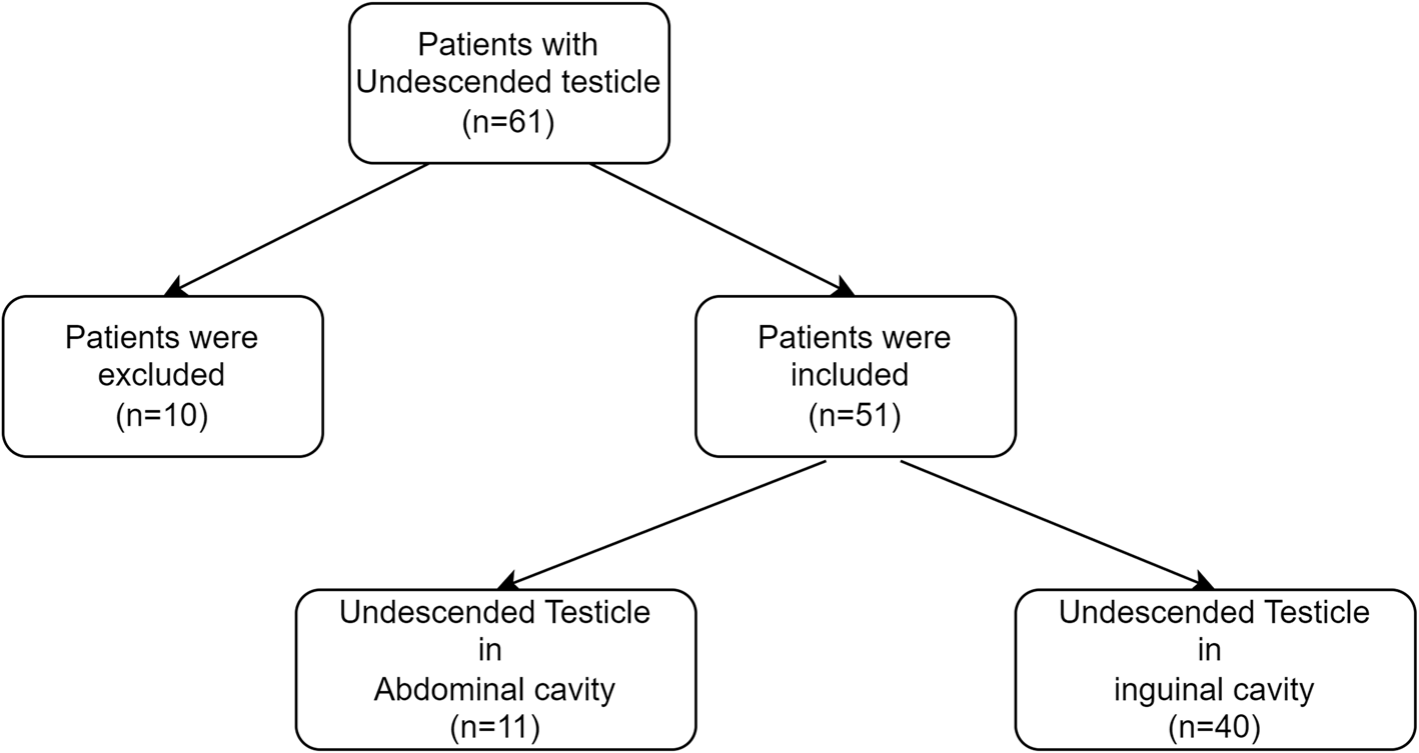
A flow diagram showing patient selection and exclusion

Patients who were admitted to our clinic suffering from an absent testicle and underwent an abdominal or inguinal orchiectomy procedure with the diagnosis of an undescended testicle after clinical evaluation were enrolled in the study. Individuals under 18 years of age or with a clinical history of medical treatment for undescended testicles were excluded, as well as the patients whose data were unavailable (Fig. 1).

### Design of the study

Data of the patients who underwent an orchiectomy procedure with the diagnosis of undescended testicle regarding age, side of the undescended testicle, testicular volume (length (L) × width (W) × height (H) × 0.52), spermatic cord length, testosterone level, semen volume, and count, the ratio of progressive motility, non-progressive motility, immotile sperms, and sperms morphology, and histopathological reports of undescended testicles were collected. who manuel for human semen analysis was used to evaluate semen samples [9].

### Histopathological examination of the orchiectomy specimens

Surgical specimens were fixed in formalin solution, embedded in paraffin wax, sectioned at 4 µm, stained using hematoxylin and eosin (H&E), and evaluated histologically by a senior pathologist. Mean seminiferous tubules diameter and thickness, germ cell count, interstitial Leydig cell count, cellular composition of tubule wall layer, and intratubular germ cell neoplasia (ITGCN) were analyzed. Critical characteristics for ITGCN were larger than normal spermatogonia and usually have a prominent irregular nucleus, distinct nucleoli, coarse clumps of chromatin, and abundant cytoplasm. If fewer than 50 tubules were in a section, all of the present tubules were examined. Otherwise, a total of 50 consecutive tubules were evaluated. Histomorphometric analyses were performed by light microscopy at a total magnification of 400x [10].

A total of 50 tubules with a round shape were selected, and tubule diameters and thicknesses were measured for each tubule using a micrometer eyepiece (Olympus, Tokyo, Japan) to calculate the mean values. The mean germ cell count per tubule was calculated in randomly selected 50 tubules. For the interstitial Leydig cell count, fifty randomly assigned intertubular areas were examined under high power. The cellular composition of the tubular wall layer was analyzed by numbering each layer as follows: number 1 for Sertoli cells, number 2 for spermatogonia cells, number 3 for spermatocyte cells, and number 4 for spermatid cells [10].

### Statistical analyses

Statistical analyses were performed using SPSS Statistics 20.0 software (SPSS Inc., Chicago, IL, USA) and Microsoft Excel computer programs. The normality hypothesis was tested by using the Shapiro–Wilk Test. Descriptive statistics were presented as median and min–max for non-normally distributed data. According to normality test results, the Mann–Whitney U test was used to compare groups. A chi-square test was used to compare categorical variables. A two-tailed *P* value < 0.05 was considered statistically significant.

## Results

A total of 51 patients, of whom 11 fell into group 1 and 40 fell into group 2, were enrolled in the study. The median age of all patients was 21 (range 20-22 years) years. Between Group 1 and Group 2, median ages did not differ significantly (*p* = 0.859). Statistical analyses between groups for median ages, testicular volumes, spermatic cord lengths, blood testosterone levels, semen volume and counts, and the ratio of progressive motility, non-progressive motility, immotile sperms, and sperms morphology were demonstrated in Table 1.

**Table 1:** Comparison of patients' general data, testicular characteristics, semen analysis results and statistical analysis of these findings. Mann–Whitney U test was used for the analysis

	*Group 1 (n=11)* *Median (Min-Max) *	*Group 2 (n=40)* *Median (Min-Max) *	*p* *value*
Age (years)	21 (20-24)	21 (20-21)	0.859
Length of the spermatic cord (mm)	30 (30-60)	40 (30-50)	0.554
Testicular volume (cm^3^)	3.12 (2.34-6.17)	7.4 (4.81-10.4)	0.002*
Testosterone level (ng/mL)	432.40 (192.48-452)	400.91 (187.79-582.09)	0.622
Semen volume (mL)	2.5 (2-3.5)	2.5 (2-3.87)	0.853
Total sperm count (10^6^)	0 (0-69)	33 (9.75-61.25)	0.306
Progressive motility (%)	0 (0-25.5)	22.35 (10.37-34.85)	0.019*
Overall motility (%)	0 (0-54.2)	43.2 (27.5-56.72)	0.50
Immotile sperm (%)	100 (45.8-100)	56.8 (43.27-72.5)	0.50
Morphology (%)	0 (0-50)	50 (6.25-55)	0.029*

*p<0.005

Median testicular volume was significantly lower in group 1 than in group 2 (*p* = 0.002). In semen analyses, the ratio of progressive motility and sperms with normal morphology was considerably higher in group 2 (*p* = 0.019 and *p* = 0.029, respectively). Other parameters did not differ significantly between groups.

Comparison of histopathological parameters such as seminiferous tubules diameters and thicknesses, cellular composition of the tubule walls, and counts of germ cells and Leydig cells between groups were shown in Table 2.

**Table 2:** Comparison of histopathological parameters such as seminiferous chareceteristics, tubule structure, cellular distribution or presence of ITGCn and statistical analysis of these findings. Mann–Whitney U test and chi-square test were used for the analysis

		*Group 1 (n=11)* *Median (Min-Max) or n (%)*	*Group 2 (n=40)* *Median (Min-Max) or n (%)*	*p* *value*
seminiferous tubules diameter (µm)		155 (127-177)	154 (124-177.25)	0.864
seminiferous tubules thickness (µm)		5.9 (4.6-7.1)	7.7 (5.7-10)	0.051
Seminiferous Tubules	Sertoli cell	20 (10-80)	25 (0-77.5)	0.254
	Spermatogonia	30 (20-50)	35 (20-57.5)	0.459
	Spermatocyte	15 (0-30)	15 (5-27.5)	0.991
	Spermatid	0 (0-10)	0 (0-17.5)	0.764
Germ cell count		10 (1-15)	9 (2.25-15)	0.671
Leydig cell count		20 (10-50)	42.5 (10-73.75)	0.420
ITGCN		3 (27.27%)	10 (25%)	0.878

There was no significant difference in histopathological parameters between groups. However, seminiferous tubules thickness was lower in those with abdominal-localized testis. Seminiferous tubules thickness showed a trend toward significance (*p* = 0.051).

The mean follow-up duration of 20 (3 patients in group 1 and 17 patients in group 2) out of 51 patients who continued routine outpatient appointments was 92.4 months. Of those, 12 (60%) were married and eight were single (40%). Of married individuals, 11 had (91.66%) offspring, and none of the male offspring of those had an undescended testicle. All single individuals had the willingness to have children. None of the patients experienced a contralateral testicular tumor in follow-ups.

## Discussion

This study, which assessed probable histopathological differences regarding the localization of the undescended testicle in patients of similar age groups, concluded with five principal endpoints: in patients with abdominal-localized testicles, mean testicular volume was lower, the blood testosterone level was higher, and seminiferous tubules were thinner, whereas, in patients with inguinal-localized testicles, the ratio of progressive motility of sperm cells and sperms with normal morphology were higher.

Elevated blood testosterone level despite the lower testicular volume in cases with abdominal-localized testicles suggests a suggestive of altered androgen signaling in this group. Lower ratios of progressive motility and sperms with normal morphology in cases who underwent an orchiectomy procedure with the diagnosis of abdominal-localized testicles might be due to seminiferous tubules pathology. It is well-known that Sertoli cells in seminiferous tubules have a regulatory role in the progression of spermatogenesis. Lower seminiferous tubules thickness in the abdominal-localized undescended testicle group compared with the inguinal-localized group might help explain the impaired semen parameters.

Testosterone and FSH (follicle-stimulating hormone) regulate spermatogenesis indirectly via Sertoli cells [11]. In patients with abdominal-localized testicles, similar cellular composition in seminiferous tubules in histopathological comparison of both testicles, in addition to elevated testosterone levels, supports the defective receptor hypothesis [12].

There is a close relationship between the seminiferous tubules structure and the development of the sperm cell. Spermatogenesis involves sequential and complicated steps in which diploid spermatogonia mature and differentiate into haploid spermatozoa in testicular seminiferous tubules. Elements that disrupt the tubule structure can lead to apoptosis and decreases in sperm cells. [13, 14] Despite the elevated testosterone level in cases with abdominal-localized testicles, semen parameters were better in the inguinal-localized group. This may be due to defective maturation, especially in the basal compartment, and impaired cellular composition in the seminiferous tubules following dysregulation of spermatogenesis, which is usually supposed to progress from the basal compartment to the luminal field.

In adult patients with an undescended testicle, the orchiectomy procedure is performed as a standard treatment against the possibility of malignancy because it does not jeopardize testosterone levels and fertility [15]. The number of studies examining undescended testicles histopathologically in adults is fewer than in children. Most of these studies included patients who underwent an orchiopexy procedure and focused on assessing the fertility potential in the following years. There may be multiple causes of infertility, including the underlying cause of testicular maldescent, preoperative anatomical position of the testis, testicular injury, and acquired disease, in addition to postoperatively recurrent epididymo-orchitis [16]. In a study examining specimens of testicular tissues from orchiopexy procedures, the authors concluded that total germ cell histopathology was not associated with hormone levels and semen parameters and could provide limited information about fertility potential in the following years [17]. In the literature, the only study with a comparable design focused on the presence of ITGCN and probable malignancies in undescended testicle specimens [8]. In our research, fertility-related parameters were affected more severely, and ratios of progressive motility and sperms with normal morphology were significantly lower in the abdominal-localized group. However, the ITGCN rate was substantially higher than the 2% level Koni and colleagues found in their study.

Testicular atrophy, age at presentation and history of cryptorchidism were independent predictors of ITGCN. Patients presenting at age 30 years or younger alsohad an increased prevalence of ITGCN (20.5% to37.5%), and 1 study reported patients age 30 oryounger had a 1.7-fold increased RR of contralateral ITGCN vs patients older than 30 years (95% CI1.2e2.6). In 1 study a history ofcryptorchidism was reported in 16.7% of patientswith ITGCN vs 8.3% of those without [18]. We interpreted that the reason why the rate of ITGCN in our study was higher than that determined in the literature could be that the patients with cryptorchidism were also young.

There are some limitations of this study. First, the retrospective single-center design of this study restricted the interpretation of the results. Second, inhibin, FSH, and luteinizing hormone levels would have contributed to more comprehensive comments on the outcomes. Third, testosterone receptor activity in the testicular tissue would have been measured using androgen receptor levels. However, our study could contribute to the literature despite these limitations.

## Conclusion

The descent of the testis in the inguinal canal is valuable for the development of the testicular tissue, especially the seminiferous tubule, supports increased sperm motility and the presence of morphologically normal sperm cells. In follow-ups of patients who underwent an orchiectomy procedure, no low rate of having offspring and the absence of undescended testicles in them could be considered as favoring results according to the patients. We think that studies with larger patient groups might provide a comprehensive understanding of the effects of the internal inguinal canal on testicles.

## Data Availability

No datasets were generated or analysed during the current study.
